# Supplementary material for: Maternal and congenital toxoplasmosis in Mayotte: Prevalence, incidence and management
Source: PLoS Negl Trop Dis. 2023 Mar 20;17(3):e0011198. doi: 10.1371/journal.pntd.0011198 (PMC10063165; doi:10.1371/journal.pntd.0011198)
Supplement: Attachment — Submitted filename: 2022-Oct 12-PNTD-D-22-01194.pdf [file pntd.0011198.s001.pdf]

PLOS Neglected Tropical Diseases

Maternal and congenital toxoplasmosis in Mayotte: prevalence, incidence and management

--Manuscript Draft--

**Manuscript Number:**	PNTD-D-22-01194
**Full Title:**	Maternal and congenital toxoplasmosis in Mayotte: prevalence, incidence and management
**Short Title:**	Maternal and congenital toxoplasmosis in Mayotte: prevalence, incidence and management
**Article Type:**	Research Article
**Keywords:**	congenital toxoplasmosis; maternal toxoplasmosis; Mayotte
**Abstract:**	Background<br><br>Toxoplasmosis is an infection caused by an intracellular protozoan, Toxoplasma gondii . It is usually asymptomatic, but toxoplasmosis acquired during pregnancy can cause congenital toxoplasmosis, potentially resulting in fetal damage. Epidemiological information is lacking for toxoplasmosis in Mayotte (a French overseas territory). We evaluated (1) the prevalence of maternal toxoplasmosis, (2) the incidence of maternal and congenital toxoplasmosis, and (3) the management of congenital toxoplasmosis in Mayotte.<br><br>Methodology / Principal Findings<br><br>We collected all the available data for toxoplasmosis serological screening during pregnancy and maternal and congenital cases of toxoplasmosis obtained between January 2017 and August 2019 at the central public laboratory of Mayotte (Mamoudzou). Using toxoplasmosis serological data from samples collected from 16,952 pregnant women we estimated the prevalence of toxoplasmosis in Mayotte at 67.19%. Minimum maternal toxoplasmosis incidence was estimated at 0.29% (49/16,952, 95% CI (0.0022-0.0038)), based on confirmed cases of maternal primary infection only. The estimated incidence of congenital toxoplasmosis was 0.09% (16/16,952, 95% CI (0.0005-0.0015). Missing data made it difficult to evaluate management, but follow-up was better for mothers with confirmed primary infection and their infants.<br><br>Conclusions / Significance<br><br>The seroprevalence of toxoplasmosis among pregnant women and the incidence of toxoplasmosis are higher in Mayotte than in mainland France. There is a need to improve the antenatal toxoplasmosis screening and prevention programme, providing better information to physicians and the population, to improve management and epidemiological monitoring.
**Additional Information:**	
**Question**	**Response**
**Financial Disclosure**<br><br>Enter a financial disclosure statement that describes the sources of funding for the work included in this submission. Review the submission guidelines for detailed requirements. View published research articles from *PLOS NTDs* for specific examples.	The funders had no role in study design, data collection and analysis, decision to publish, or preparation of the manuscript.

Powered by Editorial Manager® and ProduXion Manager® from Aries Systems Corporation

This statement is required for submission and **will appear in the published article** if the submission is accepted. Please make sure it is accurate.<br><br>**Unfunded studies**<br><br>Enter: *The author(s) received no specific funding for this work.*<br><br>**Funded studies**<br><br>Enter a statement with the following details:<br>• Initials of the authors who received each award<br>• Grant numbers awarded to each author<br>• The full name of each funder<br>• URL of each funder website<br>• Did the sponsors or funders play any role in the study design, data collection and analysis, decision to publish, or preparation of the manuscript?<br>• **NO** - Include this sentence at the end of your statement: *The funders had no role in study design, data collection and analysis, decision to publish, or preparation of the manuscript.*<br>• **YES** - Specify the role(s) played.<br><br>* typeset	
**Competing Interests**<br><br>Use the instructions below to enter a competing interest statement for this submission. On behalf of all authors, disclose any competing interests that could be perceived to bias this work—acknowledging all financial support and any other relevant financial or non-financial competing interests.<br><br>This statement **will appear in the**	The authors have declared that no competing interests exist.

**published article** if the submission is accepted. Please make sure it is accurate. View published research articles from *PLOS NTDs* for specific examples.<br><br>**NO authors have competing interests**<br><br>Enter: *The authors have declared that no competing interests exist.*<br><br>**Authors with competing interests**<br><br>Enter competing interest details beginning with this statement:<br><br>*I have read the journal's policy and the authors of this manuscript have the following competing interests: [insert competing interests here]*<br><br>* typeset<br><br>This statement is required for submission and **will appear in the published article** if the submission is accepted. Please make sure it is accurate and that any funding sources listed in your Funding Information later in the submission form are also declared in your Financial Disclosure statement.	
**Data Availability**<br><br>Authors are required to make all data underlying the findings described fully	Yes - all data are fully available without restriction

Powered by Editorial Manager® and ProduXion Manager® from Aries Systems Corporation

available, without restriction, and from the time of publication. PLOS allows rare exceptions to address legal and ethical concerns. See the PLOS Data Policy and FAQ for detailed information.<br><br>A Data Availability Statement describing where the data can be found is required at submission. Your answers to this question constitute the Data Availability Statement and **will be published in the article**, if accepted.<br><br>**Important:** Stating 'data available on request from the author' is not sufficient. If your data are only available upon request, select 'No' for the first question and explain your exceptional situation in the text box.<br><br>Do the authors confirm that all data underlying the findings described in their manuscript are fully available without restriction?	
**Describe where the data may be found in full sentences. If you are copying our sample text, replace any instances of XXX with the appropriate details.**<br><br>• If the data are **held or will be held in a public repository**, include URLs, accession numbers or DOIs. If this information will only be available after acceptance, indicate this by ticking the box below. For example: *All XXX files are available from the XXX database (accession number(s) XXX, XXX.*).<br>• If the data are all contained **within the manuscript and/or Supporting Information files**, enter the following: *All relevant data are within the manuscript and its Supporting*	All relevant data are within the manuscript and its Supporting Information files.

Information files.<br>• If neither of these applies but you are able to provide **details of access elsewhere**, with or without limitations, please do so. For example:<br><br>*Data cannot be shared publicly because of [XXX]. Data are available from the XXX Institutional Data Access / Ethics Committee (contact via XXX) for researchers who meet the criteria for access to confidential data.*<br><br>*The data underlying the results presented in the study are available from (include the name of the third party and contact information or URL).*<br>• This text is appropriate if the data are owned by a third party and authors do not have permission to share the data.<br><br>* typeset	
Additional data availability information:	

Powered by Editorial Manager® and ProduXion Manager® from Aries Systems Corporation

Click here to access/download;Manuscript;Manuscrit pour PLoS 1.docx

Maternal and congenital toxoplasmosis in Mayotte: prevalence, incidence and management

Laure Kamus[1*], Sophie Belec[2], Laurent Lambrecht[3], Soumeth Abasse[3], Sophie Olivier[4], Patrice Combe[4], Pierre-Emmanuel Bonnave[5], Christelle Vauloup-Fellous[6]

[1]Department of Medical Biology, Félix-Guyon Hospital Center, Saint-Denis, La Réunion, France

[2]Department of Obstetrics and Gynaecology, Mayotte Hospital Center, Mayotte, France

[3]Paediatric Centre, Mayotte Hospital Centre, Mayotte, France

[4]Department of Medical Biology, Mayotte Hospital Centre, Mayotte, France

[5]Mayo Bio Laboratory, Mamoudzou, Mayotte, France

[6]Universite Paris Saclay, INSERM U1193, AP-HP, Hôpital Paul Brousse, Virology Department

* Corresponding author

E-mail : laure.kamus@chu-reunion.fr

Abstract

Background: Toxoplasmosis is an infection caused by an intracellular protozoan, *Toxoplasma gondii*. It is usually asymptomatic, but toxoplasmosis acquired during pregnancy can cause congenital toxoplasmosis, potentially resulting in fetal damage. Epidemiological information is lacking for toxoplasmosis in Mayotte (a French overseas territory). We evaluated (1) the prevalence of maternal toxoplasmosis, (2) the incidence of maternal and congenital toxoplasmosis, and (3) the management of congenital toxoplasmosis in Mayotte.

Methodology / Principal Findings: We collected all the available data for toxoplasmosis serological screening during pregnancy and maternal and congenital cases of toxoplasmosis obtained between January 2017 and August 2019 at the central public laboratory of Mayotte (Mamoudzou). Using toxoplasmosis serological data from samples collected from 16,952 pregnant women we estimated the prevalence of toxoplasmosis in Mayotte at 67.19%. Minimum maternal toxoplasmosis incidence was estimated at 0.29% (49/16,952, 95% CI (0.0022-0.0038)), based on confirmed cases of maternal primary infection only. The estimated incidence of congenital toxoplasmosis was 0.09% (16/16,952, 95% CI (0.0005-0.0015). Missing data made it difficult to evaluate management, but follow-up was better for mothers with confirmed primary infection and their infants.

Conclusions / Significance: The seroprevalence of toxoplasmosis among pregnant women and the incidence of toxoplasmosis are higher in Mayotte than in mainland France. There is a need to improve the antenatal toxoplasmosis screening and prevention programme, providing better information to physicians and the population, to improve management and epidemiological monitoring.

Key words: congenital toxoplasmosis, maternal toxoplasmosis, Mayotte

Author summary:

Toxoplasmosis, a zoonotic disease caused by a ubiquitous protozoan parasite *Toxoplasma gondii* is usually asymptomatic. However, maternal infection during pregnancy can cause congenital toxoplasmosis with potentially severe neurologic or ophthalmological sequelae. Prevalence varies from country to country depending on socio-economic conditions, dietary habits and hygiene and the prevalence is higher in resource-poor countries. In Mayotte (a French overseas territory), recommendations for toxoplasmosis prevention and screening are partially applied. Indeed, many women don’t speak French, serological follow up is generally irregular and responsible for delayed introduction of treatment and consequently more congenital toxoplasmosis cases. To our knowledge, few published data were available on maternal and congenital toxoplasmosis for this territory. A better understanding of the epidemiology of this parasite would allow surveillance, information and limitation of maternal-fetal transmission to reduce the number of congenital toxoplasmosis cases and improve the management of toxoplasmosis in Mayotte.

Introduction

Toxoplasma gondii is a ubiquitous protozoan parasite that causes toxoplasmosis (TX), a zoonosis with a worldwide distribution. This intracellular parasite infects warm-blooded animals, including humans, and is estimated to have infected more than one third of the world's population [1]. Primary infection (PI) is usually (80% cases) asymptomatic in immunocompetent individuals [2, 3]. However, if a woman is infected during pregnancy, congenital TX may occur, with potentially severe sequelae (neurological and/or ocular damage) or a fatal outcome (*in utero* abortion, foetal/neonatal death) [1, 4, 5]. The risk of transmission to the foetus is closely related to gestational age at PI in the mother, ranging from <15% in the first trimester of pregnancy to almost 70% in the third trimester [6]. By contrast, congenital TX is most severe if the mother is infected during the first trimester of pregnancy [3, 5]. The rapid, early diagnosis of PI in the mother makes it possible to provide treatment (which has been shown to lower transplacental transmission rates), and specific follow-up (regular ultrasound examinations, amniocentesis and, if necessary, a change of treatment) [7, 8]. In France, it has been mandatory, since 1992, to perform monthly serological screening in seronegative women and to provide hygiene recommendations until one month after delivery [9, 10]. In 2006, a surveillance programme for maternal and congenital TX was launched in France (mainland France and overseas territories), providing valuable information annually concerning the prevalence and management of these infections [11]. Mayotte is an island that became an overseas *département* (a territorial unit similar to a county) of France in 2011; it was not included in the surveillance network until 2020 [12]. However, French recommendations for screening and prevention strategies in all pregnant women were applied in Mayotte for several years before it acquired this status.

In 2019, Mayotte had 270,000 inhabitants, with about 9,600 births recorded by the regional health agency each year. Seroprevalence in mainland France has been decreasing since the 1960s, and is currently estimated at about 30% [11, 13]. Little information about seroprevalence is available for Mayotte, and the real figures on the ground may be very different from those for mainland France, because of the tropical weather conditions, which may facilitate the survival of oocysts in the environment (hot and humid climate, long periods of rainfall) and high rates of reservoir infection (favoured by the large number of young feral cats). Moreover, this island has a high population density, with many living in precarious conditions, high rates of poverty and illiteracy, and a diet very different from that in mainland France [14]. The prevalence of TX could therefore be higher in Mayotte than in mainland France, and studies are required to determine the actual prevalence in Mayotte. Medical cover is insufficient in Mayotte (the density of doctors in Mayotte is one third that in mainland France for general practitioners, and one quarter that in mainland France for specialists) [15], and there are only two medical laboratories on the island (one private and one public laboratory). This lack of medical facilities undoubtedly has an impact on the management of pregnant women and their babies. We performed a retrospective study (from January 2017 to August 2019), to evaluate the prevalence of maternal TX in Mayotte, the incidence of maternal and congenital TX infection, and their management.

Materials and methods

Population

We collected all the TX serology results available from the central public laboratory (*Centre Hospitalier de Mayotte*, Mamoudzou, France) for pregnant women tested between January 2017 and August 2019.

Data collection

Data were collected retrospectively from the patients' medical records. The following items were recorded for the women: all TX serological/PCR results available, gestational age at maternal PI, gravidity, parity, amniocentesis/no amniocentesis, ultrasound examinations, and treatment (molecules, duration). The following items were recorded for the neonate: all TX serological/PCR results available, cerebral ultrasound and ophthalmological examinations, and treatment at birth (molecules, duration).

Biological data were collected from the database of the central laboratory of the *Centre Hospitalier de Mayotte* (InLog, *INLOG* HOLDINGS FRANCE SAS, Limonest, France). In cases of missing data during pregnancy follow-up, we obtained the missing data from the only other laboratory in Mayotte (SELARL MAYO BIO, Mamoudzou), whenever possible.

Serology and PCR

TX-IgG and IgM were performed with Access TOXO (Beckman Coulter, Tennessee, USA), and TX-IgG avidity tests were performed with VIDAS TOXO AVIDITY (BioMérieux, Craponne, France), according to the manufacturers' recommendations. High TX-IgG avidity made it possible to exclude recent PI within the last four months. In cases of low or intermediate TX-IgG avidity, it was not possible to exclude recent PI, and IgG kinetics were analysed on samples collected three to four weeks later. A doubling of TX-IgG titre was considered to confirm recent PI, whereas a stable TX-IgG titre was considered to exclude recent PI, within the last two months.

TX-PCR (on amniotic fluid and blood) was performed at a private laboratory in the Parisian region (CERBA, St Ouen l'Aumône, France).

Case definition (according to French National recommendations (9, 21, 22))

Confirmed maternal TX PI (CPI): maternal PI was considered certain if seroconversion occurred during pregnancy, or if positive results were obtained for the detection of TX-IgM and TX-IgG, with low TX-IgG avidity and a doubling of TX-IgG titre on a second sample collected three to four weeks later in women less than two months into gestation at the time of the first serological test.

Possible maternal TX PI (PPI): maternal PI was considered possible if positive results were obtained for TX-IgM and TX-IgG detection, with low or intermediate TX-IgG avidity, and a stable TX-IgG titre for a second sample collected three to four weeks later in women less than two months into gestation at time of the first serological test.

Non-excluded maternal TX PI (NEPI): maternal PI could not be excluded if positive results were obtained for TX-IgM and TX-IgG detection, with high TX-IgG avidity, in women more than four months into gestation.

Confirmed congenital TX infection (CCI): congenital TX infection was considered certain if positive results were obtained for TX-IgM detection and/or if TX-PCR results on peripheral blood were positive in the first week of life.

Statistical analysis

Univariate analysis was performed to compare the incidence of maternal TX and congenital TX between Mayotte and mainland France and/or other French overseas territories. A z-test was performed with Stata software (version 12.0), and p-values <0.05 were considered significant. A 95% confidence interval (95% CI) was calculated with the free software VassarStats: Website for statistical Computation on http://vassarstats.net/.

Results

The regional health agency (ARS) of Mayotte recorded approximately 9,600 births each year from 2017 to 2019. During our study period (January 2017 to August 2019), an estimated 25,745 infants were born alive to 25,088 women [16, 17]. We investigated 16,952 pregnancies, and our study therefore had an exhaustivity of 67.57% (95% CI: 0.67-0.68). The demographic records provided by the Mayotte ARS indicated that 17,240 infants were born to the 16,952 mothers for whom serological results were available.

Prevalence of maternal toxoplasmosis

From January 2017 to August 2019, TX-IgG tests were positive in 67.19% (11,390/16,952, 95% CI (0.66-0.68)) of pregnant women and negative in 31.77% (5,386/16,952, 95% CI (0.31-0.32)) of pregnant women. In 1.04% (176/16,952, 95% CI (0.009-0.012)) of cases TX-IgG results were equivocal, and the patients were considered seronegative in subsequent analyses. The prevalence of maternal TX in Mayotte was, therefore, 67.19%.

Incidence of maternal toxoplasmosis

During the study period, TX-IgM was detected in 272 patients, two of whom were excluded due to seroconversion during the postpartum period rather than during pregnancy.

The results for the 270 samples testing positive for TX-IgM collected from pregnant women were (Fig 1):

- 46/270 (17.78% (95% CI (0.14-0.23)) tested negative for TX-IgG, and 27/46 (58.70%) subsequently seroconverted and were considered to be cases of CPI;

- 224/270 (82.96%, 95% CI (0.78-0.87)) tested positive for TX-IgG. In 59/224 (26.34%, 95% CI (0.21-0.32)) cases, TX-IgG avidity was low. In 22/224 (9.82%, 95% CI (0.07-0.14)) cases, an analysis of TX-IgG kinetics confirmed CPI. In 6/224 cases, preconception PI was considered. Postconceptional PI was considered possible in 31/224 cases (0.14%, 95% CI (0.10-0.19)) because the first sample was collected more than two months into the pregnancy;
- 165/224 (73.66%, 95% CI (0.68-0.79)) tested positive for TX-IgG with high TX-IgG avidity. In 86/165 cases, preconceptional PI was considered. Postconceptional PI could not be excluded in 79/165 (47.88%, 95% CI (0.40-0.55)) cases, because the first sample was collected more than four months into the pregnancy.

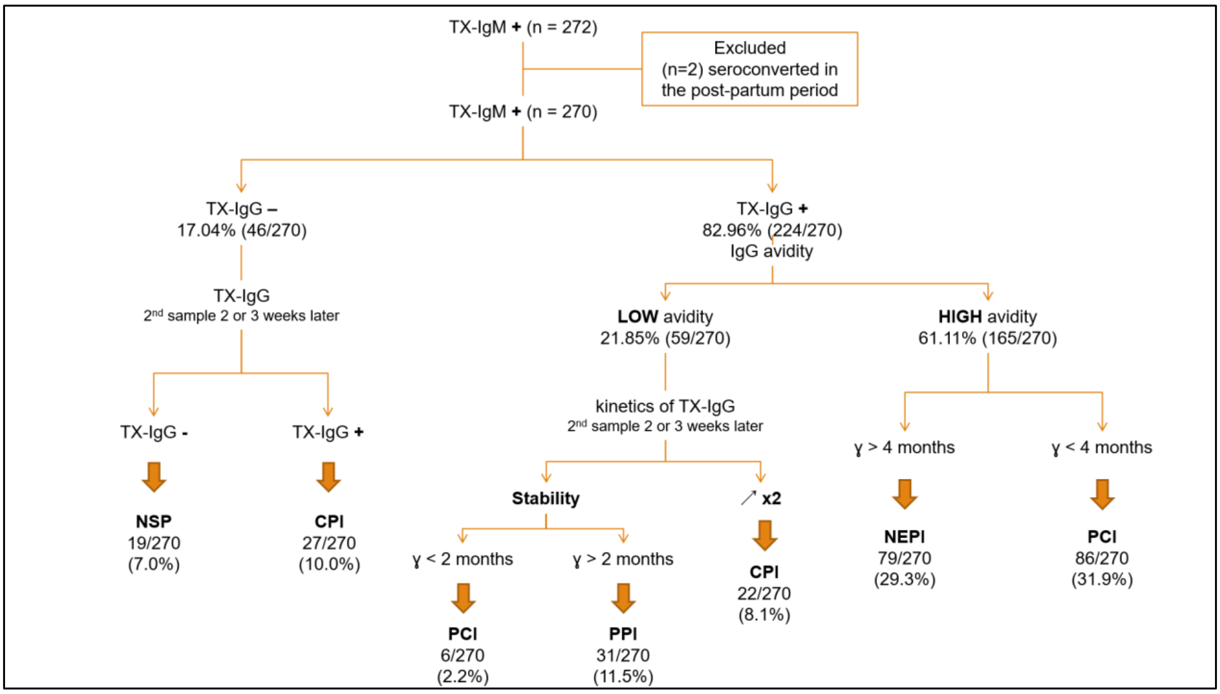

<u>**Fig 1**</u>: **Classification of maternal [illegible]oplasmosis primary infection (PI).** (ɣ: pregnancy, PCI: preconceptional PI, NEPI: PI not excluded, NSP: non-specific IgM, PPI: possible PI, CPI: confirmed PI). Classification according to the recommendations of the French National Reference Centre for Toxoplasmosis [9, 18, 19]

The minimum incidence of maternal TX (considering only CPI) was 0.29% (49/16,952, 95% CI (0.0022-0.0038)) and the maximal incidence (considering CPI and PPI) was 0.47%

(80/16,952, 95% CI (0.0038-0.0058)). These values correspond to 2.89 to 4.72/1,000 pregnant women per year. TX PI during pregnancy could not be excluded in 79/270 cases (0.29%, 95% CI (0.24-0.35) (NEPI).

Demographic information and management of maternal TX PI

Focusing on the 49 CPI, most of these cases occurred during the third trimester of pregnancy (65.31%, 32/49, 95% CI (0.51-0.77)), treatment information was not available for 53.06% (26/49, 95% CI (0.39-0.66) and only 32.65% (16/49, 95% CI (0.21-0.47)) received treatment (spiramycin, as Rovamycine®) (Table 1). Prenatal ultrasound follow-up was performed in 48.98% (24/49) cases, and abnormalities related to congenital TX were observed in 37.50% (9/24). Amniocentesis was performed in only 4.08% (2/49) of the women (Table 1). The demographic and management details for PPI and NEPI are available in table 1.

		Confirmed primary infections (CPI) (n=49)	***Possible primary infections (PPI) (n=31)***	***Non-excluded (NEPI) (n=79)***
Age		25 (16-38)	27 (15-54)	26 (15-41)
	Unknown	4/49 (8.16%)	3/31 (9.68%)	2/79 (2.53%)
Gravidity		3 (1-8)	2 (1-6)	6 (1-8)
	Unknown	22/49 (44.90%)	18/31 (58.06%)	29/79 (37.71%)
Parity		5 (1-6)	3 (1-4)	8 (1-7)
	Unknown	22/49 (44.90%)	19/31 (61.29%)	30/79 (37.97%)
Trimester of infection	1st trimester	0/49 (0.00%)	0/31 (0.00%)	1/79 (1.27%)
	2nd trimester	15/49 (30.61%)	12/31 (38.71%)	4/79 (5.06%)
	3rd trimester	32/49 (65.31%)	16/31 (51.61%)	71/79 (89.87%)
	Unknown	2/49 (4.08%)	3/31 (9.68%)	3/79 (3.80%)
Treatment	Yes	16/49 (32.65%)	3/31 (9.680%)	1/79 (1.27%)
	No	7/49 (14.29%)	0/31 (0.00%)	0/79 (0.00%)
	Unknown	26/49 (53.06%)	28/31 (90.32%)	78/79 (98.73%)
	Yes	24/49 (48.98%)	2/31 (6.45%)	1/79 (1.27%)
	Normal	15/24 (62.50%)	1/2 (50.00%)	1/1 (100.00%)

Antenatal ultrasound follow-up	Abnormal*	9/24 (37.50%)	1/2 (50.00%)	0/1 (0.00%)
	No	24/49 (48.98%)	0/31 (0.00%)	4/79 (5.06%)
	Unknown	1/49 (2.04%)	29/31 (93.55%)	74/79 (93.67%)
Amniocentesis	Yes	2/49 (4.08%)	1/31 (3.23%)	1/79 (1.27%)
	No	47/49 (95.92%)	30/31 (96.77%)	78/79 (98.73%)

Table 1: Demographic and management data for pregnant women with confirmed primary infection (CPI), possible primary infection (PPI) and non-excluded primary infection (NEPI). * Abnormalities related to congenital toxoplasmosis.

Incidence of congenital toxoplasmosis

In total, 50 infants were born to the 49 mothers with CPI. During the study period, congenital TX diagnosis was based on positive TX-IgM and/or positive TX-PCR results for a peripheral blood sample collected from the neonate. At least one serum sample was collected from most neonate (90.00% (45/50)) during the neonatal period (median: 1 day (range 1-54 days)) and TX-PCR was also performed on blood for 78.00% (39/50) (Table 2). Overall, 16/50 (32.00%) children had CCI (Fig 2).

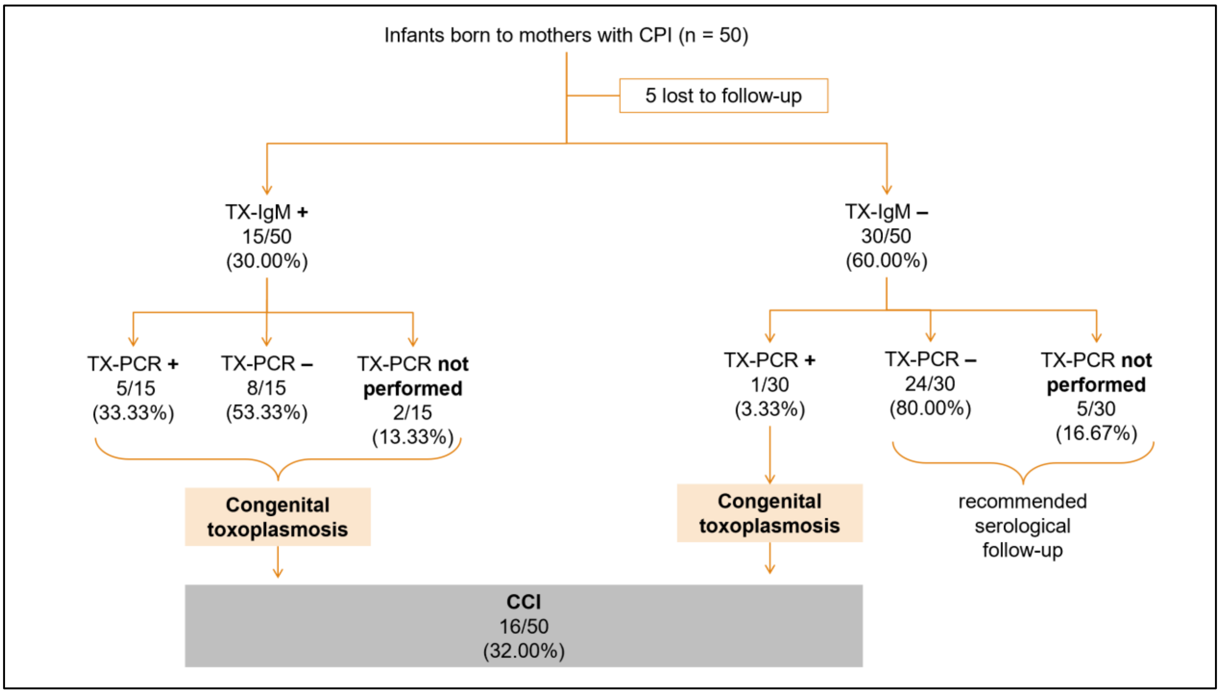

Fig 2: Diagnosis algorithm for congenital toxoplasmosis. PCR: polymerase chain reaction, CCI: confirmed congenital infection

In total, 31 infants were born to the 31 mothers with PPI, and 80 were born to the 79 mothers with NEPI. Only 9/111 children underwent at least one serological test and 12/111 underwent TX-PCR on blood (Table 2).

		Infants born to mothers with CPI (n=50)	***Infants born to mothers with PPI (n=31)***	***Infants born to mothers with NEPI (n=80)***
Serological tests	Yes	45/50 (90.00%)	7/31 (22.58%)	2/80 (2.50%)
	No	5/50 (10.00%)	0/31 (0.00%)	1/80 (1.25%)
	Unknown	0/50 (0.00%)	24/31 (77.42%)	77/80 (96.25%)
PCR	Yes	39/50 (78.00%)	9/31 (29.03%)	3/80 (3.75%)
	No	11/50 (22.00%)	0/31 (0.00%)	4/80 (5.00%)
	Unknown	0/50 (0.00%)	22/31 (70.97%)	73/80 (91.25%)
Treatment	Yes	10/50 (20.00%)	1/31 (3.23%)	0/80 (0.00%)
	No	2/50 (4.00%)	0/31 (0.00%)	1/80 (1.25%)
	Unknown	38/50 (76.00%)	30/31 (96.77%)	79/80 (98.75%)
Imaging (antenatal ultrasound)	Yes	25/50 (50.00%)	2/31 (6.45%)	1/80 (1.25%)
	No	24/50 (48.00%)	0/31 (0.00%)	4/80 (5.00%)
	Unknown	1/50 (2.00%)	29/31 (93.55%)	75/80 (93.75%)

Table 2: Biological data, treatment and imaging information for infants born to women with confirmed primary infection (CPI), possible primary infection (PPI) or non-excluded primary infection (NEPI). Two sets of twins were born, one to a mother with CPI and the other to a mother with NEPI.

Given the large numbers of missing data for children born to women with PPI and NEPI, only a minimum incidence of congenital TX was calculated, based on the demographic records of children born to women with CPI. We estimated that 17,240 infants were born to the 16,952 mothers with available serological results during the study period. The minimal incidence was, therefore, 9.28/10,000 liveborn infants (16/17,240, 0.09% (95% CI (0.0005-0.0015)).

Management of newborns

For infants born to mothers with CPI, treatment information was available for 24.00% (12/50) cases and imaging data were available for 98.00% (49/100). More than 90.00% of the data were not reported in medical records (serological tests, PCR, treatment and imaging data) for infants born to mothers with PPI or NEPI (Table 2).

Less than half the infants with CCI (43.75%, 7/16) underwent eye fundus examination, and the results were normal in all cases (Table 3). More than half underwent transfontanelle cerebral ultrasound examinations (62.50%, 10/16), the results of which were normal in 80.00% of cases (8/10) (Table 3). Cerebral abnormalities were reported in two cases: a persistence of microcysts in the caudate nucleus in one infant, and small subependymal haemorrhages typical of toxoplasmosis in the second infant, with no calcifications or cerebral ventricle dilation in either case.

			Confirmed congenital infections (TX-IgM + and/or PCR +) (n=16)
Serological tests and PCR	Yes		16/16 (100.00%)
	No		0/16 (0.00%)
	Unknown		0/16 (0.00%)
Antenatal ultrasound	Yes		10/16 (62.50%)
		Normal	6/10 (60.00%)
		Abnormal*	4/10 (40.00%)
	No		6/16 (37.50%)
	Unknown		0/16 (0.00%)
Trans-fontanelle cerebral ultrasound scan	Yes		10/16 (62.50%)
		Normal	8/10 (80.00%)
		Abnormal*	2/10 (20.00%)
	No		2/16 (12.50%)
	Unknown		4/16 (25.00%)
	Yes		7/16 (43.75%)

Eye fundus examination	Normal	7/7 (100.00%)
	Abnormal*	0/7 (0.00%)
	No	0/16 (0.00%)
	Unknown	9/16 (56.25%)
Follow-up > 1 month	Yes	5/16 (31.25%)
	No	0/16 (0.00%)
	Unknown	11/16 (68.75%)

Table 3: **Management of newborns with confirmed congenital infection (CCI).** * Abnormalities related to congenital toxoplasmosis. (Imaging abnormality: one case with small subependymal haemorrhages typical of toxoplasmosis (no calcification and no ventricular dilation) confirmed on check-up imaging and one case of persistent microcysts in the caudate nucleus)

Discussion

Thanks to several national prevention programmes, the prevalence of maternal TX infection has steadily decreased over the last few decades in mainland France. Indeed, it fell from 84% in 1960 to 33.7% in 2016 [9, 12, 20-25]. A similar decrease has been reported for French overseas territories, despite less data being available. The first studies in French overseas territories in 1995 reported a seroprevalence of 61.4%, which decreased to 42.9% in 2016 (French West Indies, French Guiana, La Reunion) [20, 24, 25]. TX prevalence increases with age and depends on geographic location, socioeconomic level, eating habits and cultural practices. In our study, in Mayotte, we found a maternal TX seroprevalence of 67.19%, a value similar to that reported for mainland France in the 1980s and higher than the values for other French overseas territories in 2016. One of the main factors underlying this difference is probably diet, particularly as concerns the consumption of meat and contaminated vegetables. Toxoplasmosis prevalence is lower in countries in which meat is eaten well cooked (United Kingdom, North America). It is slightly higher in mainland France, where meat is often eaten raw or smoked [9]. In Mayotte, meat is also often eaten raw or smoked, or cooked on a barbecue, which does not destroy toxoplasmosis cysts. Another important factor influencing prevalence

is socioeconomic conditions, including, in particular, access to clean water, and proximity to animals (especially feral cats) that can serve as major reservoirs of *T. gondii*. Hot and humid climatic conditions also favour the spread of toxoplasmosis [26, 27]. One of the strengths of our study is its exhaustivity of about 67% for the 2017/2019 period. However, prevalence may well be underestimated in this study, as at least 20% of women in Mayotte are thought to illegal immigrants, with an even lower socioeconomic status, and many of these women may not have undergone serological testing at all during their pregnancy.

The incidence of TX during pregnancy varies with prevalence. The incidence of TX during pregnancy is well-documented in mainland France, due to the mandatory serological screening programme. This incidence has decreased over time, reaching a predicted 1.6/1,000 in 2020 [8, 21]. In Mayotte, we found the incidence of maternal TX infection to be 2.89 to 4.72/1,000 per year. This higher incidence of maternal TX is consistent with the prevalence of 67.19%, and is significantly different from that of mainland France (*p*-value <0.05). However, this rate is probably underestimated due to the large number of cases that could not be formally classified. Indeed, one of the limitations of our study is the inconsistency of serological follow-up in pregnant women. In many cases, only one serological test was performed during pregnancy, often more than four months after conception. This lack of follow-up is due to local factors, such as a lack of awareness in women of child-bearing age, the late diagnosis of pregnancy and frequent misunderstanding of the French language (French is the mother tongue of only 10% of the mothers in Mayotte). This last factor is almost certainly also a major reason for the lack of awareness concerning behaviours at risk for toxoplasma infection and for women not following primary toxoplasmosis prevention rules.

For the management of maternal TX, our study highlights a greater acceptance of non-invasive examinations, such as ultrasound scans, rather than amniocentesis (in women with CPI particularly: 49.98% (24/49; 95% CI (0.36-0.63) and 4.08% (2/49; 95% CI (0.01-0.14), respectively). PCR on amniotic fluid is an excellent tool for confirming foetal infection and ensuring optimal management of the child at birth, particularly in terms of reducing unnecessary treatment with possible adverse effects [28, 29]. Several studies have reported a risk of pregnancy loss of <1% during amniocentesis procedures [30]. The poor acceptance of amniocentesis in Mayotte is a serious obstacle to antenatal and neonatal diagnosis, representing a loss of opportunity in the management of this disease. It is probably related to cultural and religious factors. Providing women with better information about the disease, the various diagnostic tests and their benefits would raise awareness, potentially increasing the acceptability of amniocentesis.

The worldwide incidence of congenital TX ranges from 1 to 30/10,000 live-born infants [9, 31]. In mainland France, the incidence is about 2 to 3 cases/10,000, and this rate has remained stable since 2007 [11, 12]. In Mayotte, this incidence was estimated at 9.28/10,000 liveborn infants, which is three times the rate in mainland France (p-value <0.05). This difference may, of course, be due to the higher incidence of maternal TX, but it may also reflect a late introduction of treatment due to irregular and/or delayed serological follow-up, which concerns more than just TX. One limitation of our results was the significant number of missing data, but this lack of information may suggest that treatments were not prescribed or not taken by pregnant women. Most infants born to mothers with PPI and NEPI were not explored, suggesting that the incidence reported here may be an underestimate. In new-borns, TX-IgM tests were performed at the age of three days (mean: 2.87 days; median: 1 day; range: 0-43 days), in accordance with French recommendations [9]. Overall, we report better follow-up for

infants born to mothers with CPI, other than for treatment, which was poorly documented (76% missing data). Exploration of the immune responses of the mothers and infants by immunoblotting can improve the accuracy of congenital TX diagnosis, thereby facilitating early management [32]. Such screening was not available in Mayotte at the time of our study, but was implemented soon after.

Given the seriousness of this disease, several countries have, like France, implemented preventive programmes. For example, in Italy, Denmark, and Germany, congenital TX monitoring has been performed since 1997, 1999 and 2001, respectively [33]. However, in 2007, Denmark decided to stop its screening programme, because of the high costs relative to the low level of potential benefit. Other European countries, such as Austria, Lithuania, and Slovenia, consider congenital TX to be a public health problem and deliver advice about its prevention, but have yet to establish an effective congenital TX surveillance system. Austria and Brazil, like France, have TX screening programmes [34], but screening is not recommended in the United Kingdom and North America, where TX incidence is low [35-37]. A prevention programme including the monthly screening of all seronegative women has been implemented in France since 1978, with the systematic declaration of congenital TX cases since 1992. The prevention programme implemented in 2010 has proved effective in mainland France, decreasing the levels of epidemiological markers and rates of severe forms of TX [11]. Unfortunately, this programme is not fully applied in Mayotte, where serological screening for TX is performed during pregnancy (when women attend prenatal consultations), but congenital TX cases are not reported to the TX NRL (national reference laboratory). Several factors contribute to this situation: the frequent use of false identities by illegal immigrants (20% of pregnant women in Mayotte are thought to be illegal immigrants), the difficulties understanding French of most of the women and a lack of awareness of surveillance programmes among

healthcare workers, together with challenging working conditions (insufficient numbers of healthcare staff) [38].

Our findings have important public health and social implications and should lead to the strengthening of educational programmes in Mayotte, for both healthcare practitioners and pregnant women. Specific cultural characteristics and practices should be considered as potentially important independent determinants in the planning of further counselling strategies, as a better understanding of complementary diagnostic examinations and the consequences of the disease would improve patient awareness and decrease opposition to specific management practices, including treatment and invasive procedures.

This study is the first to report epidemiological data on maternal and congenital TX management in Mayotte and highlights possibilities for improvements, although many issues remain to be addressed.

Acknowledgements

We thank Dr. Guillaume Miltgen (UMR Processus Infectieux en Milieu Insulaire Tropical, CNRS 9192, INSERM U1187, IRD 249, Université de La Réunion, Saint-Denis, La Réunion, France) for comments on the manuscript and for his insights in review and editing.

References

1. Moncada PA, Montoya JG. Toxoplasmosis in the fetus and newborn: an update on prevalence, diagnosis and treatment. Expert Review of Anti-infective Therapy. 2012;10(7):815-28. doi: 10.1586/eri.12.58.

2. Association française des enseignants de p. Parasitoses et mycoses des régions tempérées et tropicales / ANOFEL, Association française des enseignants et praticiens hospitaliers de parasitologie et mycologie médicales. 6e édition ed. Issy-les-Moulineaux: Elsevier-Masson; 2019.

3. Montoya JG, Liesenfeld O. Toxoplasmosis. The Lancet. 2004;363(9425):1965-76. doi: https://doi.org/10.1016/S0140-6736(04)16412-X.

4. Jones JL, Lopez A, Wilson M, Schulkin J, Gibbs R. Congenital toxoplasmosis: a review. Obstet Gynecol Surv. 2001;56(5):296-305. Epub 2001/05/03. doi: 10.1097/00006254-200105000-00025. PubMed PMID: 11333376.

5. Thiébaut R, Leproust S, Chêne G, Gilbert R. Effectiveness of prenatal treatment for congenital toxoplasmosis: a meta-analysis of individual patients' data. The Lancet. 2007;369(9556):115-22. doi: 10.1016/s0140-6736(07)60072-5.

6. Wallon M, Peyron F, Cornu C, Vinault S, Abrahamowicz M, Kopp CB, et al. Congenital Toxoplasma infection: Monthly Prenatal Screening Decreases Transmission Rate and Improves Clinical Outcome at Age 3 Years. Clinical Infectious Diseases. 2013;56(9):1223-31. doi: 10.1093/cid/cit032.

7. Fricker-Hidalgo H, Brenier-Pinchart MP, Schaal JP, Equy V, Bost-Bru C, Pelloux H. Value of Toxoplasma gondii detection in one hundred thirty-three placentas for the diagnosis of congenital toxoplasmosis. Pediatr Infect Dis J. 2007;26(9):845-6. Epub 2007/08/28. doi: 10.1097/INF.0b013e318123e8d3. PubMed PMID: 17721384.

8. Mandelbrot L, Kieffer F, Wallon M, Winer N, Massardier J, Picone O, et al. Toxoplasmose pendant la grossesse : proposition actuelle de prise en charge

pratique. Gynécologie Obstétrique Fertilité & Sénologie 2021. doi: https://doi.org/10.1016/j.gofs.2021.03.003.

9. Haute Autorité de Santé HAS. Diagnostic biologique de la toxoplasmose acquise du sujet immunocompétent (dont la femme enceinte), la toxoplasmose congénitale (diagnostic pré- et postnatal) et la toxoplasmose oculaire. Evaluation des technologies de santé. Saint-Denis La Plaine: 2017.

10. Journal Officiel de la République Française. Decree 92-143 on mandatory premarital and postnatal examinations. 14 February 1992 ed1992.

11. Villena I, Ancelle T, Delmas C, Garcia P, Brézin AP, Thulliez P, et al. Congenital toxoplasmosis in France in 2007: first results from a national surveillance system. Eurosurveillance. 2010;15(25). doi: 10.2807/ese.15.25.19600-en.

12. Toxoplasmose Cndrdl. Rapport annuel d'activité 2020 sur l'exercice de 2019. 2020.

13. Picone O, Fuchs F, Benoist G, Binquet C, Kieffer F, Wallon M, et al. Toxoplasmosis screening during pregnancy in France: Opinion of an expert panel for the CNGOF. Journal of Gynecology Obstetrics and Human Reproduction. 2020;49(7):101814. doi: https://doi.org/10.1016/j.jogoh.2020.101814.

14. Carme B, Tirard-Fleury V. La toxoplasmose chez la femme enceinte en France : séroprévalence, taux de séroconversion et niveau de connaissance des mesures préventives. Tendances 1965–1995. Médecine et Maladies Infectieuses. 1996;26:431-6. doi: https://doi.org/10.1016/S0399-077X(96)80188-0.

15. Bouet P, Gerard-Varet J-F. Atlas de la démographie médicale en France: Situation au 1er janvier 2020. Conseil national de l’ordre des médecins, 141p. 2020.

16. REPEMA. Panel 2018 : Indicateurs de santé périnatale à Mayotte. 2019.

17. INSEE. Bilan démographique 2018 à Mayotte - Insee flash Mayotte. 2019.

18. Villard O, Cimon B, L'Ollivier C, Fricker-Hidalgo H, Godineau N, Houze S, et al. Serological diagnosis of Toxoplasma gondii infection: Recommendations from the French National Reference Center for Toxoplasmosis. Diagn Microbiol Infect Dis. 2016;84(1):22-33. Epub 2015/10/16. doi: 10.1016/j.diagmicrobio.2015.09.009. PubMed PMID: 26458281.

19. Villard O, Jung-Étienne J, Cimon B, Franck J, Fricher-Hidalgo H, Godineau N, et al. Sérodiagnostic de la toxoplamose en 2010: conduite à tenir et interprétation en fonction des profils sérologiques obtenus par les méthodes de dépistage. 2010.

20. Tourdjman M TC, De Valk H, Goulet V, Le Strat Y. 2015. Toxoplasmose chez les femmes enceintes en France : évolution de la séroprévalence et des facteurs associés entre 1995 et 2010, à partir des Enquêtes nationales périnatales. . Bull Epidémiol Hebd. 2015;(15-16):264-72. .

21. Nogareda F, Le Strat Y, Villena I, De Valk H, Goulet V. Incidence and prevalence of Toxoplasma gondii infection in women in France, 1980–2020: model-based estimation. Epidemiology and Infection. 2014;142(8):1661-70. doi: 10.1017/s0950268813002756.

22. Papoz L, Sarmini H, Funes A, Comiti V. Étude de la prévalence de l’empreinte immunologique de la rubéole, de la toxoplasmose, du cytomégalovirus, de l’herpes et de l’hépatite B chez 8594 femmes de 15 a45 ans en France en 1982–1983. BEH. 1984;20:2-3.

23. Martins M, Desmont G, Couvreur J, Ben Rachid M. Toxoplasmosis, the mother and the child. Arch Fr Pediatr. 1965;22:1183-90.

24. Berger F, Goulet V, Le Strat Y, Desenclos JC. Toxoplasmosis among pregnant women in France: Risk factors and change of prevalence between 1995 and 2003. Revue d'Épidémiologie et de Santé Publique. 2009;57(4):241-8. doi: https://doi.org/10.1016/j.respe.2009.03.006.

25. Robinson E, de Valk H, Villena I, Le Strat Y, Tourdjman M. National perinatal survey demonstrates a decreasing seroprevalence of Toxoplasma gondii infection among pregnant women in France, 1995 to 2016: impact for screening policy. Euro Surveill. 2021;26(5). Epub 2021/02/06. doi: 10.2807/1560-

7917.Es.2021.26.5.1900710. PubMed PMID: 33541484; PubMed Central PMCID: PMCPMC7863230.

26. Rostami A, Riahi SM, Esfandyari S, Habibpour H, Mollalo A, Mirzapour A, et al. Geo-climatic factors and prevalence of chronic toxoplasmosis in pregnant women: A meta-analysis and meta-regression. Environ Pollut. 2021;288:117790. Epub 2021/07/31. doi: 10.1016/j.envpol.2021.117790. PubMed PMID: 34329056.

27. INSEE. Synthèse démographique, sociale et économique. 2019.

28. de Oliveira Azevedo CT, do Brasil PE, Guida L, Lopes Moreira ME. Performance of Polymerase Chain Reaction Analysis of the Amniotic Fluid of Pregnant Women for Diagnosis of Congenital Toxoplasmosis: A Systematic Review and Meta-Analysis. PLoS One. 2016;11(4):e0149938. Epub 2016/04/08. doi: 10.1371/journal.pone.0149938. PubMed PMID: 27055272; PubMed Central PMCID: PMCPMC4824461.

29. Mandelbrot L, Kieffer F, Sitta R, Laurichesse-Delmas H, Winer N, Mesnard L, et al. Prenatal therapy with pyrimethamine + sulfadiazine vs spiramycin to reduce placental transmission of toxoplasmosis: a multicenter, randomized trial. Am J Obstet Gynecol. 2018;219(4):386.e1-.e9. Epub 2018/06/06. doi: 10.1016/j.ajog.2018.05.031. PubMed PMID: 29870736.

30. Salomon LJ, Sotiriadis A, Wulff CB, Odibo A, Akolekar R. Risk of miscarriage following amniocentesis or chorionic villus sampling: systematic review of literature and updated meta-analysis. Ultrasound Obstet Gynecol. 2019;54(4):442-51. Epub 2019/05/28. doi: 10.1002/uog.20353. PubMed PMID: 31124209.

31. Varella IS, Canti IC, Santos BR, Coppini AZ, Argondizzo LC, Tonin C, et al. Prevalence of acute toxoplasmosis infection among 41,112 pregnant women and the mother-to-child transmission rate in a public hospital in South Brazil. Mem Inst Oswaldo Cruz. 2009;104(2):383-8. Epub 2009/05/12. doi: 10.1590/s0074-02762009000200037. PubMed PMID: 19430669.

32. Jost C, Touafek F, Fekkar A, Courtin R, Ribeiro M, Mazier D, et al. Utility of immunoblotting for early diagnosis of toxoplasmosis seroconversion in pregnant women. Clin Vaccine Immunol. 2011;18(11):1908-12. Epub 2011/08/31. doi: 10.1128/CVI.05303-11. PubMed PMID: 21880851.

33. Bénard A, Petersen E, Salamon R, Chêne G, Gilbert R, Salmi LR, et al. Survey of European programmes for the epidemiological surveillance of congenital toxoplasmosis. Eurosurveillance. 2008;13(15). doi: 10.2807/ese.13.15.18834-en.

34. Rorman E, Zamir CS, Rilkis I, Ben-David H. Congenital toxoplasmosis--prenatal aspects of Toxoplasma gondii infection. Reprod Toxicol. 2006;21(4):458-72. Epub 2005/11/29. doi: 10.1016/j.reprotox.2005.10.006. PubMed PMID: 16311017.

35. Chapple J, editor Antenatal screening for Toxoplasmosis External review against criteria set by the UK National Screening Committee (UK NSC)2015.

36. Paquet C, Yudin MH. No. 285-Toxoplasmosis in Pregnancy: Prevention, Screening, and Treatment. J Obstet Gynaecol Can. 2018;40(8):e687-e93. Epub 2018/08/15. doi: 10.1016/j.jogc.2018.05.036. PubMed PMID: 30103893.

37. Montoya JG, Remington JS. Management of Toxoplasma gondii infection during pregnancy. Clin Infect Dis. 2008;47(4):554-66. Epub 2008/07/16. doi: 10.1086/590149. PubMed PMID: 18624630.

38. Bouet P, Mourgues J-M. Atlas de la démographie médicale en France: Situation au 1er janvier 2018. Conseil national de l'ordre des médecins, 165p. 2018.
