# Supplementary material for: Maternal and congenital toxoplasmosis in Mayotte: Prevalence, incidence and management
Source: PLoS Negl Trop Dis. 2023 Mar 20;17(3):e0011198. doi: 10.1371/journal.pntd.0011198 (PMC10063165; doi:10.1371/journal.pntd.0011198)
Supplement: Attachment — Submitted filename: review answers.docx [file pntd.0011198.s002.docx]

**Responses to the review**

**METHODS**

**Reviewer 1**: satisfactory

**Reviewer 2**:

- Is the population clearly described and appropriate for the hypothesis being tested :

*=> See comments below on the need to better define the population of pregnant women in terms of length of pregnancy monitored.*

🡺 The answers below.

- Are there concerns about ethical or regulatory requirements being met?

*=> no*

🡺 The study was conducted in accordance with the Declaration of Helsinki. General informed consent for the use of their anonymous clinical information was obtained prospectively from all subjects involved in the study as soon as pregnancy management is totally or partially performed in Mayotte Hospital Center.

**Reviewer 3**:

- Are there concerns about ethical or regulatory requirements being met?

=> *Authors can provide ethical clearance or permission (though this is a retrospective study)*

🡺 The study was conducted in accordance with the Declaration of Helsinki. General informed consent for the use of their anonymous clinical information was obtained prospectively from all subjects involved in the study as soon as pregnancy management is totally or partially performed in Mayotte Hospital Center.

**RESULTS**

**Reviewer 1** :

- Please denote the sex of infants born to mothers with toxo. It is important because maternal inflammation could have more adverse impacts on males.

🡺 Thank you for your comment. Among the 16 newborns with confirmed congenital infection 8 were males and 8 were females. Information added in the text.

- Please add clinical information (symptoms and outcomes) of infected mothers or infants if available.

🡺 Thank you for your comment but unfortunately these data are not available.

**Reviewer 2** :

- See below for comments on the results section.

🡺 Answers on the results section.

**Reviewer 3** :

- Are the figures (Tables, Images) of sufficient quality for clarity? Yes. In addition, an infographic figure of the overall study is recommended.

🡺 We believe that the addition of a new figure, as suggested by the reviewer, would be repetitive with the 2 figures presented in the manuscript showing how the selection and diagnosis was performed. Maybe reviewer could tell us what he would like to see in this figure? We have a graphical abstract suggested below:

**CONCLUSIONS**

**Reviewer 1** : satisfactory

**Reviewer 2** :

- See below for comments on statement provided in the discussion

🡺 Answers on the discussion section.

**Reviewer 3** : No question

**Editorial and Data Presentation Modifications**

**Reviewer 1** : (No Response)

**Reviewer 2** :

*General comments:*

It is current practice to use “Toxoplasma infection” instead of “Toxoplasmosis” for cases that are not patent.

“Tx” is not a standard abbreviation for “Toxoplasma infection” or “Toxoplasma gondii” and no single abbreviation should be used for the infection and the agent causing it. (When referring to IgG we would say “anti Toxoplasma” IgG and not “toxoplasmosis IgG”) Its extensive use throughout the manuscript largely decreases its readability. Congenital Toxoplasma infection can be abridged with CT, which will reduce the number of characters to such extend as to allow the use of "infection" instead of "PI" in pregnant women. In the context of the study, it is obvious that primary infections are the events of interest rather than reactivations

🡺 Changes in abbreviations have been made throughout the manuscript.

The abbreviation CPI, PCI, PPI, NPI should be simplified.

🡺 CPI for confirmed maternal primo infection was replaced by confirmed toxoplasmosis

🡺 PPI for possible maternal primo infection was replaced by (Possible *toxoplasma* infection)

🡺 NEPI for non-excluded maternal primo infection was replaced by non-excluded

🡺 CCI for confirmed congenital infection was replaced by CT

*Introduction*

The use of brackets should be avoided for important information about treatment and follow up.

🡺 Brackets have been removed (Line 80-82).

Hygiene recommendations are well placed since 1983. The French surveillance programme does not include registering maternal infections, but only CT.

🡺 Thank you for this clarification, indeed the recommendations are older. The modification of the manuscript has been done (Line 82-84 & Line 86).

The exact dates of the study period do not need to be given here (there are given in the methods section).

🡺 We agree with your pertinent remark which allows to lighten the manuscript (Line 109).

*Material and methods*

- **Line 113-115**: It is standard practice when dealing with a population of pregnant women to define the inclusion criteria based on their dates of pregnancy or of delivery.

It does not seem to have been the criteria used here. If the entry point for patients' inclusion was the existence of at least one serological test for anti Toxoplasma IgG and IgM collected within a given period, it should be stated more clearly. "All pregnant women for whom at least one anti serological test for anti toxoplasma IgG and IgM was performed between January 2017 and August 2019" were included regardless of the gestational age at sampling.

🡺 Thank you for your comment. We have changed the wording of the inclusion criteria to be more precise. (Line 116-118).

- **Lines 117-127**: as mentioned earlier, replace TX with Toxoplasma

🡺 Changes in abbreviations have been made throughout the manuscript.

- **Lines 130-156**: Tx can be deleted throughout because of the mention of the tests: ex: Vidas Toxo Avidity. The decision to consider equivocal IgG results as negative has to be defined here and not in the results section.

🡺 Abbreviations TX have been delated.

🡺 A sentence has been added in the MM section concerning equivocal IgG (Line 137-138).

- **Lines 135-137**; information about doubling of IgG titres.. should be removed (repetition with the next paragraph)

🡺 This information was indeed accidentally repeated, this sentence has been deleted.

- **Lines 141 156**: this paragraph is very hard to read because of the many repeated words. It also seems that some situations are missing (criteria for excluding an infection during pregnancy).

I would suggest limiting to three situations that involve different management.

- No infection during pregnancy: past infection (Early and stable IgG) or still negative at delivery
- Proven Infection during pregnancy: true seroconversion or doubling of IgG in the context of high titters
- All other situations including: late positive IgG, insufficient screening until delivery.

🡺 We understand your comment and therefore simplify some abbreviations by deleting NEPI in favor of "non-excluded" and simplify as recommended CCI for confirmed toxoplasmosis cases by CT.

However, for the distinction between maternal and possible infections we wish to keep this information. Indeed, serological follow-up of pregnant women being challenging in Mayotte, it is not always obvious to confirm toxoplasmosis diagnosis. We have chosen to carry out our calculations according to this distinction, in particular the evaluation of maximum and minimum incidences, allowing us to highlight difficulties of toxoplasmosis diagnosis in Mayotte.

- How prevalence was measured in pregnant women should be mentioned here = proportion of pregnant women with IgG above the cut off at their first test

🡺 It is notified in case definition section for seroprevalence (Line 167-169).

- The same goes for the incidence of Toxoplasma infection in pregnancy. It should be stated that only true seroconversions and proven profils of acute infection were taken into account to estimate incidence. What was the denominator used: what is the total number of pregnant women regardless of the length of pregnancy covered by the screening, or the total number of month of pregnancy covered by the screening, if no tests were performed at delivery.

🡺 It is notified in case definition section for incidence (Line 168-172).

- How incidence of Congenital toxoplasmosis was computed should also be stated here rather on **line 240 and 242**

🡺 We thank you for your remark, it is notified (Line 177-178).

*Results*

- The results section is overall difficult to read because of the information that should be given in the material and methods section, because confirmed cases of infection tend to be presented in the same length of details as non confirmed cases (including in table 1 and 2), and because of the use of many abbreviations.

🡺 Most of the abbreviations have been removed to clarify. Concerning the distinction between confirmed and unconfirmed cases, we believe that it is necessary to show this difference, which highlights the challenging follow-up of pregnant women in Mayotte.

- Figure 1 is especially hard to read because of the abbreviations. CPI, NEPI and PCI should best be written in clear, and because it mixes criteria for the different profiles, findings at the first test, and findings of follow up. I would suggested limiting figure 1 to findings at the first test.

🡺 Figures 1 and 2 have been reworked for more clarity.

- Tables are also difficult to read because of the repetition of the column denominator on each line. "%" does not need to be repeated on each line if define in the column head.

🡺 Changes have been made in the various tables for more readability. (Table 1, 2, 3)

- "Primary" can be excluded from Table 1.

🡺 Primary was excluded

- The number of tests per pregnancy should be given at the beginning of the section, and

🡺 We appreciate the reviewer’s insightful suggestion; however, this information was not available. That is why we mention a minimum and maximum incidence for toxoplasmosis infection.

- how many seronegative patients were tested until delivery. It is a key information, to estimate the likelihood of detecting an acute infection during the whole course of pregnancy.

🡺 We understand this comment. This information was not collected and was hardly recoverable. The objective was to evaluate the incidence which is inevitably underestimated because of the non-optimal screening of pregnant women.

- **Line 173**: Prevalence of maternal toxoplasmosis should be replaced with Prevalence of IgG and IgM in pregnant women.

🡺 We thank the reviewer for pointing this out. However, we have defined our seroprevalence calculation with the maternal toxoplasmosis notion and we would like to keep this initial definition for clarity in the manuscript.

- It should be stated that it was measured on the first test, performed on average at x weeks of pregnancy (+ range)

🡺 Prevalence was calculated on the first test available but sometimes only one serology was performed during pregnancy and sometimes quite late in pregnancy. This is why we cannot provide this data and believe it will not be very informative.

*Discussion*

- **Line 265**. The decrease in prevalence rather reflects epidemiological changes and rarer opportunities of encounters with the parasites as they are also detected in men.

🡺 Indeed this aspect is not negligible in the reduction of the prevalence. Information has been added at the beginning of the introduction

- **Line 288**. This should be reworded because the risk for a single non immune pregnant woman of getting infected does not depend on the prevalence among pregnant women.

🡺 "Prevalence" refers to the overall prevalence of toxoplasmosis in the general population. Precision has been added in the manuscript. (Line 309-310)

- It also should be clearly stated that incidence among pregnant women is only based on estimates, based on modelling.

🡺 This precision is indeed important, we added this information to the manuscript (Line 315-316).

- **Line 340**. Austria monitors the total number of children recognized as infected.

🡺 Indeed, a correction has been made.

- **Line 344**. 1978 was the year when pre-marital testing was implemented. Monthly re-testing was implemented in 1992. The French Register for Congenital toxoplasmosis was only started years later. No prevention program has been implemented in 2010. This paragraph has to be rewritten.

🡺 We agree and have updated, paragraph has been rewritten (Line 365-369).

**Reviewer 3** : (No Response)

**Summary and General Comments**

**Reviewer 1** :

Overall, the manuscript is interesting; the methods are satisfactory and results are important. However, it needs to add some information of the patients:

-    Please denote the sex of infants born to mothers with toxoplasmosis. It is important because maternal inflammation could have more adverse impacts on males.

-    Please add clinical information (symptoms and outcomes) of infected mothers or infants if available.

**Reviewer 2** :

This study provides valuable information

- on the risks of maternal and congenital Toxoplasma infections in a territory and geographical area for which information is lacking

- and on the lack of compliance with the screening and management recommendation that apply to all French territories.

It clearly illustrates the overall importance of supporting the setting up of preventive actions by appropriate organization and communication, and of monitoring its long term benefits.

Care should be taken to make the information easier to read and understand by reducing the number of abbreviation and trimming unnecessary words. Strengthening the material and methods section by providing all required information will help the reader and make the results more visible.

**Reviewer 3** :

The study on "Maternal and congenital toxoplasmosis in Mayotte: prevalence, incidence and management" from France in generally interesting and provide insightful data and future direction.

- **Strengths:**

For epidemiological stand point, this study provides important public health and social implications to strengthening of educational programs (counselling strategies, complementary diagnostic examinations, and awareness) for both healthcare practitioners and pregnant women in Mayotte-France.

- **Weakness:**

one of the limitations of the study is the inconsistency of serological follow up in pregnant women.

My other comments are on the manuscript file (highlighted in yellow)

*Materials and methods :*

- Any proof of ethical or permission to conduct this study though this is a retrospective study?
- It would be more interesting if authors can provide an infographic figure of the overall study for scientific values and better understanding.
- Inclusion and Exclusion criteria of this study?

🡺 Changes made to text (Line 121-122)

*Results :*

- Figure 1: ,« .... toxoplasmosis with primary infection? »

🡺 Changes in the text

*Discussion*:

- **Line 301** : *Toxoplasma* (italic)

🡺 Changes in the text

- **Line 366** : What are these issues?

🡺 In light of your comment, sentence has been reworded (Line397-399).

*References*

🡺 We thank the reviewer for pointing this out. Changes made in the text.
